# *JAK2* V617F Genotype Is a Strong Determinant of Blast Transformation in Primary Myelofibrosis

**DOI:** 10.1371/journal.pone.0059791

**Published:** 2013-03-26

**Authors:** Giovanni Barosi, Valentina Poletto, Margherita Massa, Rita Campanelli, Laura Villani, Elisa Bonetti, Gianluca Viarengo, Paolo Catarsi, Catherine Klersy, Vittorio Rosti

**Affiliations:** 1 Unit of Clinical Epidemiology and Center for the Study of Myelofibrosis, IRCCS Policlinico S. Matteo Foundation, Pavia, Italy; 2 Biotechnology Research Area, IRCCS Policlinico S. Matteo Foundation, Pavia, Italy; 3 Unit of Clinical Immunology, Immunohematology, and Transfusion Service, IRCCS Policlinico S. Matteo Foundation, Pavia, Italy; 4 Service of Biometry and Statistics, IRCCS Policlinico S. Matteo Foundation, Pavia, Italy; UT MD Anderson Cancer Center, United States of America

## Abstract

**Purpose:**

The influence of *JAK2* V617F mutation on blast transformation (BT) and overall survival (OS) in primary myelofibrosis (PMF) is controversial. In a large cohort of patients we applied competing risks analysis for studying the influence of *JAK2*V617F mutation on BT in PMF.

**Patients and Methods:**

In 462 PMF–fibrotic type patients (bone marrow [BM] fibrosis grade >0) we computed the incidence of BT and death in the framework of Cox regression analysis and of Fine and Gray competing risks analysis for BT.

**Results:**

At the Cox regression analysis, having either a wild-type (wt) or a homozygous *JAK2*V617F genotype were factors for BT (HR, 1.98 and 2.04, respectively, with respect to the heterozygous genotype), but not for OS. At the competing risks regression analysis, the risk for BT in wt and homozygous V617F patients increased with respect to Cox analysis, giving a sHR of 2.17 and 2.12, respectively. Correcting the results for the variables that could have influence on BT, *JAK2*V617F wt and homozygous genotypes remained independently associated with BT. In a validation cohort of 133 independent cases with PMF-prefibrotic type (BM fibrosis grade  = 0), the BT predictive model including *JAK2*V617F genotype and older age retained high discriminant capacity (C statistics, 0.70; 95% CI, 0.47 to 0.92).

**Conclusion:**

The accumulation of mutated alleles in the *JAK2*V617F clone or the selective acquisition of a proliferative advantage in the wt clone are two relevant routes to BT in PMF. The influence of these results on treatment decisions with anti-JAK2 agents should be tested.

## Introduction

The somatically acquired Janus Kinase 2 (*JAK2*) mutation (V617F) is borne by approximately 60% of patients with primary myelofibrosis (PMF) [1]–[3]. The mutation increases JAK2 kinase activity, with the potential to affect phenotype and clinical outcome of mutated subjects. Accordingly, PMF V617F mutants result associated with higher white-blood cell counts, higher propensity to develop large splenomegaly, and are less likely to require transfusion during follow-up [2], [4]. At variance, different results have been obtained as far as the influence of *JAK2*V617F mutation on the major disease outcomes, such as blast transformation (BT) and overall survival (OS). The majority of the studies [3], [5]–[9] did not identify *JAK2*V617F mutational status as an independent prognostic factor for either OS or BT. Conversely, Campbell and coworkers reported that *JAK2*V617F mutated patients had poorer OS than non mutated patients [4], and Tefferi and coworkers claimed that low allele burden of *JAK2*V617F was the major determinant for BT and OS [10]. Furthermore, we documented that the presence of a *JAK2*V617F hematopoietic clone was significantly associated with BT, but not death for any cause [2], while Guglielmelli and coworkers pointed out that low allele burden at diagnosis represented an independent factor associated with shortened OS but not BT [11], [12].

In this study, we reconsidered the influence of *JAK2*V617F mutation on the outcomes of PMF, under the reasoning that the discrepancies in the published results could be determined by cohort- or design-dependent reasons. Since BT is mostly a late event in the natural history of PMF, with an actuarial median time to event of 201 months [11], the much shorter follow-up of the published case series, ranging from 23 to 38 months [2], [4], [6], [7], [11], [12], could potentially influence the results by lowering the study statistical power. Moreover, the occurrence of BT may be influenced by the risk of dying for other causes before BT occurs; thus, testing the association of *JAK2*V617F mutation with BT without taking into account this competing event could strongly influence the results [13].

From these premises, we considered our cohort of patients with PMF-fibrotic type consecutively registered from 1990 and prospectively followed, who reached a median follow-up of 3.5 years and we used statistical analysis to account for competing risks. With this cohort of patients we strove to provide a more informative population and appropriate analysis for studying the influence of *JAK2*V617F mutation on BT and OS in PMF.

## Methods

### Ethics Statement

The study was approved by the IRCCS Policlinico S. Matteo Foundation's institutional review board. Written informed consent was obtained from each patient before data were entered in the data-base.

### Study Cohorts

The study cohorts were composed by patients seen from 1990 to 2011 in our centre who received a diagnosis of PMF according to the WHO criteria [14], and in which we performed *JAK2*V617F mutation assay on peripheral blood granulocytes at diagnosis or during the follow-up. The patients' groups were obtained after a recent systematic revision of bone marrow (BM) biopsies taken at diagnosis in all cases referred to our Center for a myeloproliferative neoplasm (MPN). The first cohort (learning sample) was composed by consecutive patients with a diagnosis of PMF-fibrotic type (BM fibrosis grade 1 or more [15]). The second cohort (validation sample) was composed by consecutive patients with a diagnosis of PMF-prefibrotic type (BM fibrosis grade 0 or less than 1). Information about the patients was collected in a retrospective fashion at the time of first visit, and prospectively thereafter. All patients were characterized by the IPSS risk category [3].

### *JAK2*V617F Genotyping

DNA was extracted with standard procedures after isolation of total peripheral blood granulocytes by density gradient centrifugation. The mutational status for *JAK2* V617F was determined using both allele specific-PCR and restriction enzyme-based assay [16]. Digestion of PCR products with *Bsa*XI allowed for an estimation of the ratio between mutated and wild type alleles. Samples were scored as homozygous if the proportion of the mutant allele was >50%. A proportion of the mutant allele <50% has been defined as heterozygous/mixed clonality by Steensma et al [17]; for simplicity, such samples were scored as heterozygous. In selected patients, measurement of *JAK2*V617F allele burden was also performed using an allele-specific quantitative PCR assay which provides a sensitivity for the mutant alleles detection of 1% [18].

### Study Design and Statistical Analysis

In this longitudinal study, the primary outcome measures considered were occurrence of BT and death for any cause. A diagnosis of BT was defined as persistent elevation of PB or bone marrow blasts of 20% or more [19]. In the primary analysis, the distribution for overall and BT-free survival was estimated using the method of Kaplan and Meier (KM). Cox regression was used to test for differences in survival between groups. Hazard ratios (HR) and 95% confidence intervals were computed (95% CI). Competing risk analysis was used to compute BT cumulative incidence rates, considering non–BT mortality as the competing event. The Fine and Gray competing risks regression was used to study the determinants of BT [20]. Subhazard ratio (sHR) and 95% CI were computed. In this analysis, conventional risk factors measured at diagnosis, including age, sex, hemoglobin, white-blood cell count, percentage of blasts in PB, CD34+ cells in PB, and IPSS risk categories were analyzed. In multivariable regression, non collinear variables resulting associated with BT with p<0.1 at univariable analysis, and with a frequency of missing values lower than 20%, were used to assess whether *JAK2*V617F status independently predicted BT. Due to low number of chromosomal analyses, we did not consider karyotype information in the prediction analysis of disease outcomes. A time dependent Cox model was fitted to assess the role of JAK2 status changes during follow-up. We validated the obtained model of BT in an independent series of patients with PMF-prefibrotic type by Harrel C discrimination statistics in which the higher value is representative of better system performance. Results were considered statistically significant when 2-sided P values were less than 0.05. All computations were performed with STATA 12 (Stata Corporation, College Station, TX, USA).

## Results

### *JAK2* V617F Genotypes and Outcome Analysis in PMF-Fibrotic Type Patients

A total of 462 patients having PMF-fibrotic type were genotyped for *JAK2* V617F mutation. They were 302 men (65.4%) and the median age was 54.7 years (range, 6 to 90 years). IPSS risk distribution was 50% low, 21%, intermediate 1, 18.5%, intermediate 2, and 10.5% high. At the initial examination, 284 patients harbored a *JAK2*V617F mutation giving a frequency of the mutation of 61.5% (95% CI, 57.1% to 65.9%). One hundred and sixty-one had a heterozygous mutation (34.8%; 95% CI, 30.4% to 39.1%), while 123 (26.6%; 95% CI, 22.6% to 30.6%) had a homozygous mutation.

We prospectively collected multiple DNA samples from 244 patients (*JAK2*V617F wt, 105, *JAK2*V617F heterozygous, 83, *JAK2*V617F homozygous, 56). Different time points of the disease were analyzed for an overall follow-up of 638 patient-years. The follow-up analysis was initially done with the allele specific PCR. When a V617F genotype transition was documented, it was verified by quantitative PCR assay. No patient who initially was *JAK2*V617F wt became V617F mutated, while 3 who were V617F heterozygous became homozygous (3.6%), and 4 others became wt (4.8%). Transition from heterozygous to wt genotype was associated with the development of BT in 3 out of 4 patients. Eleven of the patients who initially were homozygous became heterozygous (19.6%). This conversion was secondary to splenectomy in one case, allogeneic stem cell transplantation in another. In eight other cases, the conversion occurred during therapy with hydroxyurea. One further patient regressed progressively from homozygous to heterozygous and to wt genotype; in this case the change in genotype was associated with the appearance of increasing number of blasts in PB up to BT.

Over a median follow-up of 42 months, 83 patients died (17.9%), 45 were lost to follow-up (9.7%), and 98 developed BT (21.2%). The median OS of the population of patients was 248 months (20.6 years); the median BT-free survival was 184 months (15.3 years). Sixty four out of 98 (65.9%) patients with BT died. Eight others were enrolled in a program of allogeneic stem cell transplantation and they are still alive. The median time from the diagnosis of BT to death was 15 months.

**Figure 1:**
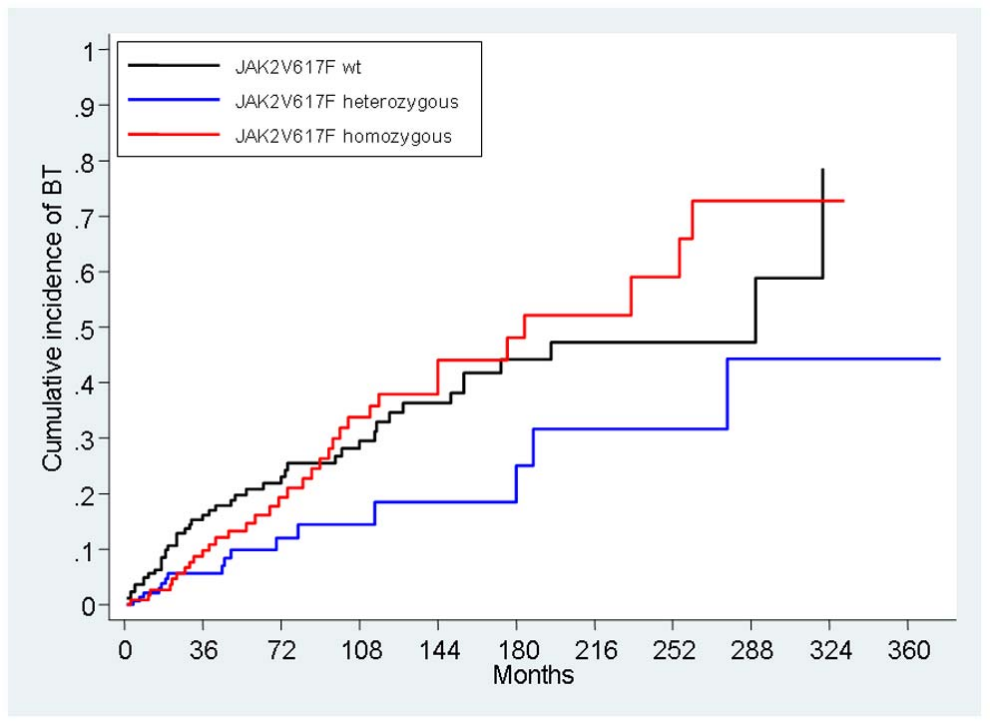
Kaplan Meier estimate of cumulative probability of blast transformation (BT) in the three genotypes for *JAK2*V617F mutation.

With the KM analysis, the risk of death for any cause was not different between the three genotypes of *JAK2*V617F mutation (P = 0.23). On the contrary, the initial status of the *JAK2*V617F genotype was significantly associated with the risk of incurring into BT (P<0.001); it resulted higher in patients who had either wt or homozygous V617F genotype with respect to those who had heterozygous genotype (HR, 1.98; 95% CI, 1.11 to 3.53; P = 0.019, and HR 2.04; 95% CI, 1.12 to 3.71; P = 0.018, respectively) (Figure 1). The median time to BT was shorter in *JAK2*V617F homozygous than in those wt (144 vs. 290 months, respectively), but the difference was not statistically significant.

**Figure 2:**
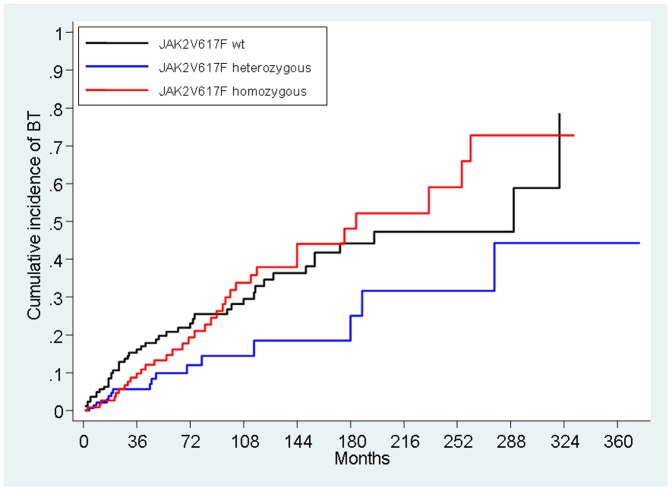
Cumulative incidence of blast transformation (BT) in the three genotypes for *JAK2*V617F mutation accounting for the competing risk of death for non-BT cause.

With the Fine and Gray model, with BT the event of interest and non-BT mortality the competing risk, the cumulative incidence of BT at the end of follow-up was 66% (95% CI, 51% to 78%) while the cumulative incidence of death was 26% (95% CI, 13% to 40%). When refining the assessment of *JAK2*V617F status at diagnosis for the risk of BT, we showed an increase in risk for wt and homozygous genotypes with respect to heterozygous, with sHR for wt, 2.17, 95% CI, 1.22 to 3.85; P = 0.008, and sHR for homozygous, 2.12, 95% CI, 1.20 to 3.75; P<0.009. sHR of the combined homozygous and wt vs. heterozygous genotype was 2.65; 95% CI, 1.56 to 4.50; P<0.001 (Figure 2).

When the sHR of BT was adjusted for the resulting risk factors for BT, the outcome resulted independently predicted by having either homozygous or wt *JAK2*V617F genotype and by older age at the initial assessment (Table 1).

**Table 1:** Regression model for competing risks (event of interest, blast transformation) in patients with primary myelofibrosis.

	Fine and Gray Model	
	sHR (95% CI)	P-value
**Univariable analysis**		
Age >50	2.29 (1.50–3.51)	<0.001
Female sex	1.41 (0.93–2.15)	0.11
Hemoglobin level	0.92 (0.80–0.98)	0.013
Leukocyte count	1.00 (0.97–1.03)	0.88
PB blasts >1%	1.17 (1.06–1.29)	0.001
CD34+ cells in PB >50×10^6^/L	2.12 (1.03–4.33)	0.040
IPSS prognostic score	1.89 (1.51–2.67)	<0.001
*JAK2*V617F wt or homozygous vs. heterozygous	2.16 (1.27–3.67)	0.004
**Multivariable analysis**		
Age>50	2.26 (1.41–3.63)	0.001
*JAK2*V617F wt or homozygous vs. heterozygous	2.48 (1.43–4.30)	0.001

Finally, change in *JAK2*V617F genotype during follow-up was associated with an increased risk of BT, which however did not reach statistical significance (HR 2.45, 95% CI, 0.89–6.71; P = 0.08) in a bivariable analysis including *JAK2*V617F status at diagnosis.

A total of 147 patients were genotyped with a quantitative assay for *JAK2*V617F mutation in granulocyte DNA collected within 6 months from diagnosis, provided that no treatment had been delivered in the meantime. A total of 97 patients were *JAK2*V617F mutated, giving an overall frequency of 65.9%. The median value of V617F allele burden was 47% (range, 6–100%). The distribution of mutated patients according to the V617F allele burden corresponded to overall frequencies of 15.5% in the first quartile, 40.2% in the second, 28.8.% in the third, and 15.5.% in the fourth. Comparing the four groups, the time to BT, death for any cause, and death for BT among the 4 quartiles were not significantly different (P = 0.5). However, there was a shorter time to BT in the upper quartile compared with lower quartiles (P = 0.04).

### Risk Assessment in the Validation Cohort of PMF-Prefibrotic Type Patients

A total of 133 patients having PMF-prefibrotic type were genotyped for *JAK2*V617F mutation, and they were chosen as validation sample. They were 52 men (39.1%) and the median age was 39 years (range, 6 to 79 years). IPSS risk distribution was 89% low, 8.1%, intermediate 1, 2%, intermediate 2, and 0.9% high. At the initial examination, 86 patients harbored a *JAK2*V617F mutation giving a frequency of the mutation of 64.6% (95% CI, 56.5% to 72.7%). Sixty-three had a heterozygous mutation (47.3%; 95% CI, 38.8% to 55.8%), while 23 (17.3%; 95% CI, 10.8% to 23.7%) had a homozygous mutation. The cumulative risk of BT at 10 years of follow-up was computed to 0% in *JAK2*V617F heterozygous patients, 18.2% (95% CI, 4.9% to 55.3%) in homozygous, and 8.4% (CI, 2.1% to 30.6%) in wt patients.

When the accuracy of BT predictive model developed from the learning sample of patients with PMF-fibrotic type, including JAK2V617F wt or homozygous genotype and age older than 50 years, was assessed on this new set of patients, the model retained its good discrimination (Harrel C statistics, 0.71; 95% CI, 0.65 to 0.77, and 0.70; 95% CI, 0.47 to 0.92, in the learning and testing sample respectively).

## Discussion

By studying a large series of patients with PMF-fibrotic type and a median follow-up of more than four years, we documented that *JAK2*V617F homozygous and wt genotype conferred higher risk of BT than having a heterozygous genotype. By a competing event model, the estimated risk of BT for homozygous and wt mutants increased with respect to the risk estimated with conventional KM regression analysis, supporting the hypothesis that competing events reduce the incidence of BT and hamper the analysis of risk factors. The risk remained statistically significant after adjustment for other risk factors for BT in PMF, such as older age, lower hemoglobin, circulating blasts and IPSS prognostic score. Moreover, the documentation that V617F homozygous mutant patients had higher risk than heterozygous was confirmed in a subpopulation of patients genotyped with a quantitative assay for *JAK2*V617F mutation, where the highest quartile had an increased risk of BT with respect the lower quartiles. With this study, however, we did not find any association between *JAK2*V617F genotype and OS.

In this study we documented that both heterozygous and homozygous genotypes are dynamic characteristics evolving spontaneously or by the effect of therapy. In a time-dependent risk analysis, we revealed that the genotype changes had a minor influence on the risk of BT. However, since V617F genotype variations during the course of the disease occurred only in few patients, this result is not conclusive on the influence that more active therapies could exert on the propensity for BT by producing genotype variations.

To validate our results, we assessed the BT prediction model obtained in the learning cohort of PMF patients in a population of PMF-prefibrotic type patients, a sample independently collected during the same period of time in our center. In this population, the prognostic model has a C statistics of 0.70, indicating that the model discriminates well the validation data. This result speaks in favor of generalizability of the proposed model, indicating a prominent role of the *JAK2*V617F genotype in the determination of BT in PMF. A limitation of our analysis is that the development and validation patients came from the same center. Efforts are underway to further validate the model on patients from other institutions.

Many reasons may justify the difference of our results from those previously reported [3], [5]–[12]. The high number of cases, the long follow-up, and the competing risks analysis are probably the most influential. However, as we have discussed in a previous paper that attained at the same series of cases [14], the unusual clinical characteristics of our patients, who showed long survival and low risk of death for non-leukemic causes, could have contributed to enlighten the mechanisms of leukemogenesis that are hidden in other series.

The result that homozygosity for *JAK2*V617F is a risk factor for BT in PMF parallels the finding that accumulation of *JAK2* mutated alleles contributes to transformation into myelofibrosis of patients with polycythemia vera [21]. Both results corroborate a progression model for MPNs according which *JAK2*V617F mutation determines genetic instability and damages which allow disease transformation [22], [23]. Different mechanisms were demonstrated able to promote additional genetic damages to *JAK2*V617F mutated cells. Plo et al. documented that *JAK2*V617F may deregulate homologous recombination which is responsible for both the loss of heterozygosity of JAK2 and the acquisition of additional genetic events [24]. Zhao et al. documented that Bcl-XL deamination in response to DNA damage is inhibited in *JAK2*V617F-positive primary cells, providing a mechanism for aberrant survival of DNA-damaged cells [25]. Dawson et al. documented that *JAK2*V617F produced phosphorylation of histone H3 tyrosine 41 residue (H3Y41) excluding heterochromatin protein 1α and promoting expression of oncogenes such as LMO2 [26]. Finally, Nakatake et al. documented that *JAK2*V617F promotes La-dependent increased translation of MDM2 mRNA which decreases p53 tumor suppression protein [27].

Concurrently, this study provides the apparently opposite evidence that PMF patients not bearing the *JAK2*V617F mutation have an approximately equal risk to evolve into BT than those with a *JAK2*V617F homozygous genotype and higher that those with heterozygous genotype. This result corroborates the evidence of clonal dominance of cells with the wt genotype reported in this work and elsewhere [6], [8], [28], [29] by the *JAK2-*mutated patients in which an entirely new JAK2 wt clone takes over the disease process by clonal selection [30].

The higher propensity to BT of the *JAK2*V617F wt malignant clone(s) with respect to the heterozygous clone may be interpreted as the occurrence of selective molecular events in V617F wt cells that provide them with high pressure for acute leukemia conversion. Evidence in favor to molecular events selective for JAK2 wt genotype exists for MPL, ASXL1, and CBL mutations that have been reported to be highly expressed in *JAK2*V617F non mutated patients with respect to those mutated [31]–[33]. The same evidence has been reported in a study of allelic imbalances associated with BT in MPNs, in which a gain of chromosomal material at 8q 24.21, a region including MYC gene, was almost exclusively found in *JAK2*V617F wt samples, suggesting that increased activity of MYC might allow selection of clones that do not require the JAK2 mutation [6]. However, for most of the known mutations occurring in the chronic phase of PMF, evidence for such a selectivity is lacking. As a matter of fact, gene mutations in TET2 [34], EZH2 [12], [35], IDH [35], or genomic changes that may induce genetic instability in PMF [6], do not entail statistical relationship with the *JAK2*V617F mutation. Thus, the pressure to BT in cases with *JAK2*V617F wt genotype is conceivably determined by the acquisition of not yet discovered, selective genetic lesions in the JAK2 wt clone which promote a clonal expansion that is higher than that in the JAK2 mutated clone.

Collectively, the results of this paper are in favor of a two routes model of BT in PMF, in which oncogenic events provide a leukemogenic potential both to *JAK2*V617F wt clone and to the *JAK2*V617F mutated clone with a homozygous genotype. These results re-open the issue of prognostic indicators for BT in PMF [36], and should be considered in the light of the current therapeutic strategies for PMF. The possibility to functionally reduce the *JAK2*V617F activity with new anti-JAK2 agents [37] questions whether these therapies, when applied in patients with high *JAK2*V617F allele burden, could prevent the evolution toward BT. On the other hand, if clonal selection is a mechanism of progression of the JAK2 wt clone, therapies that might preferentially suppress the mutant clone could favor the expansion of coexisting wt clones with subsequent BT of the latter [28]. These hypotheses deserve to be tested by trials whose experimental design includes BT as a primary end-point.
